# New insights into Sti1/Hop's cochaperone function highlight the complexity of proteostatic regulation

**DOI:** 10.1111/febs.70108

**Published:** 2025-04-21

**Authors:** Gregory Lloyd Blatch, Adrienne Lesley Edkins

**Affiliations:** ^1^ The Vice Chancellery The University of Notre Dame Australia Fremantle Australia; ^2^ Biomedical Biotechnology Research Unit (BioBRU), Department of Biochemistry, Microbiology and Bioinformatics Rhodes University Makhanda South Africa

**Keywords:** Hop, Sti1, STIP1

## Abstract

Sti1/Hop is a cochaperone that regulates Hsp70 and Hsp90 chaperones. Sti1/Hop function is perceived as limited to scaffolding chaperone complexes, although recent studies suggest a broader function. Rutledge *et al.* show that while Sti1/Hop functions within chaperone complexes under basal conditions, during high stress, it operates independently to sequester soluble misfolded protein in the cytoplasm, a function typically associated with chaperones rather than cochaperones. Furthermore, the localisation and levels of Sti1/Hop are finely tuned to ensure orderly sequestration and resolution of misfolded proteins. These data support a role for Sti1/Hop as a cochaperone specialised for stressed proteostasis networks.

## Graphical abstract

Sti1/Hop functions extend beyond scaffolding in the Hsp70‐Hsp90 chaperone complex to sequester misfolded proteins during cell stress. Consequently, imbalances between levels of Sti1/Hop relative to the rest of the proteostasis machinery negatively affect fitness and stress resilience.

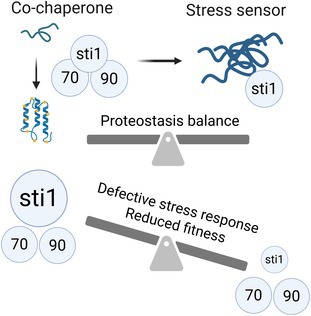

**Abbreviations**
Hop: Hsp70‐Hsp90 organising protein
Hsp: heat shock protein
JDP: J domain protein
Sti1: stress‐induced protein 1
STIP1: stress‐induced phosphoprotein 1
TPR: tetratricopeptide repeat

Protein homeostasis (proteostasis) requires that the interconnected and dynamic processes of translation, protein folding, modifications, translocation and degradation are regulated to ensure cell survival. Molecular chaperones are a class of proteins critical for maintaining protein homeostasis. Chaperones function as catalysts for the protein folding process, allowing translated peptides, particularly those with multiple domains, to achieve their inherent three‐dimensional native conformation on a biological timescale in a crowded cellular environment [1]. Many chaperones function as molecular machines undergoing dramatic conformational changes driven by energy from ATP hydrolysis to catalyse protein folding. Chaperone function is required for physiological and *de novo* protein folding, and different chaperones regulate discrete phases of protein folding. The physiological requirements for chaperone function are heightened in response to stress, where integrated chaperone networks are necessary for stress resilience [1]. However, chaperone networks need to be dynamic and responsive. Chronic, static higher order chaperone complexes, known as epichaperomes, may be hijacked to meet the increased proteostatic demands of diseases such as cancer and neurodegeneration [2].

Optimal proteostasis requires that molecular chaperone function is open to regulation. This is controlled by a cohort of regulatory proteins known as cochaperones [3]. A unified definition of a cochaperone is challenging, but the term is usually associated with an accessory protein that interacts with a chaperone to regulate chaperone function by regulating ATP binding or hydrolysis, delivering client proteins or by modulating intermediate conformations [3]. Usually, cochaperones lack independent chaperone function, although there are exceptions. Cochaperones outnumber chaperones, expanding and finetuning the same chaperone for different functions under the control of the temporal and cell‐specific expression and distribution of cochaperones [3].

The Hsp90 chaperone complex is one of the best studied chaperone networks. Prokaryotic Hsp90 (HtpG) is interesting at least in part because it lacks any known cochaperones, while in eukaryotes, Hsp90 has the most expanded and diverse cochaperone cohort [3]. This plethora of cochaperones is critical for specialising Hsp90's functions and triages the chaperone between protein folding, conformational regulation and protein degradation functions in eukaryotes. Hsp90 cochaperones are adapted for distinct binding sites on the chaperone for selected client protein families and have discrete mechanisms of action [3].

Sti1/Hop is an early‐stage Hsp70‐Hsp90 cochaperone. It is a modular protein containing three tetratricopeptide repeat (TPR) domains that differ in binding affinity for molecular chaperone partners [4]. Hsp70 is bound with high affinity at TPR1, while Hsp90 binds most strongly to TPR2A. The function of the third TPR domain, TPR2B, is somewhat perplexing. TPR2B functions as a low‐affinity binding site for Hsp70/Hsp90 peptides but can bind other nonchaperone proteins such as tubulin [4]. For many years, Sti1/Hop was considered essential as a physical bridge required to bring Hsp70 and Hsp90 into complex and therefore critical in the folding cascade [4]. However, it is clear now that the protein folding functions of Hsp90 in both prokaryotes and eukaryotes can be triaged by other cochaperones (e.g., the J domain proteins, JDPs, also called Hsp40s) [5] or achieved in the absence of Hsp90 cochaperones through direct interaction with Hsp70 [6, 7]. In the latter case, in a mechanism conserved in both prokaryotes and eukaryotes, Hsp90 releases stalled Hsp70‐client intermediates to permit spontaneous client protein folding [8]. An alternative viewpoint is that cochaperones such as Sti1/Hop are needed for the more complex proteostatic networks that only exist in eukaryotes [1]. Indeed, Sti1/Hop is exclusively found in eukaryotes; no orthologues have been identified in bacteria [1, 4]. Therefore, Sti1/Hop may be needed to specialise Hsp90 in eukaryotes towards client protein stabilisation and conformational regulation, rather than just protein folding. Indeed, in structural models of Hsp90 complexes, Sti1/Hop directly contacts the client protein [9]. However, a central role in protein stabilisation is not consistent with studies in mammalian cell line models, where Sti1/Hop can be knocked out with a minor impact on viability and relatively few changes in the global proteome [6, 10]. Sti1/Hop is not essential in yeast, *C. elegans*, or *Drosophila*, although knockout does result in reduced growth and lower stress resilience (Table 1) [11, 12, 13]. In contrast, Sti1/Hop is essential in the parasites *Leishmania donovani* and *Plasmodium falciparum*, and in the mouse (Table 1) [14, 15, 16]. Nevertheless, evidence is emerging that Sti1/Hop may have a more central role in proteostasis than was previously envisaged. For example, studies on a protein involved in multiple neurodegenerative diseases, Tar DNA‐binding protein 43 (TDP‐43), revealed that moderate expression of Sti1/Hop reduced TDP‐43 toxicity, while high expression or knockout of Sti1/Hop increased TDP‐43 toxicity [17]. Indeed, Sti1/Hop was shown to regulate the toxicity of TDP‐43 in a dose‐dependent manner [17]. These findings suggest that Sti1/Hop function is more enigmatic than originally thought and that the levels of Sti1/Hop relative to other chaperones and cochaperones are crucial to a potential central role in proteostasis; and this is supported by the recent study from Rutledge *et al*. [18]. This study suggests an alternative and more direct proteostasis role for Sti1/Hop, particularly during high protein misfolding stress conditions. Indeed, compelling evidence is provided that Sti1/Hop has Hsp90‐independent functions and can sequester soluble misfolded protein into cytoplasmic foci during proteostatic stress; a function typically associated with molecular chaperones rather than cochaperones.

**Table 1:** Cellular responses to altered Sti1/Hop abundance.

Species	Change	Phenotype	References
*Caenorhabditis elegans*	Depletion	Viable but with reduced lifespan, reduced heat tolerance, reduced fertility during stress	[11]
*Saccharomyces cerevisiae*	Deletion	Viable, but show growth defects in response to stress and accumulation of misfolded protein foci in cytoplasm	[12, 18]
	Overexpression	Viable, but with reduced fitness in response to stress	[18]
*Drosophila melanogaster*	Mutation	Viable, but reduced growth and smaller cell size	[13]
Mouse	Knockout	Embryonic lethal; impaired heat shock response (HSR)	[16]
Human cell lines	CRISPR‐mediated knockout	Viable, some growth defects and minimal changes in global proteome	[6]
	shRNA mediated depletion	Viable but with reduced growth, impaired HSR reducing stress resilience	[10]
*Leishmania donovani*	Knockout	Essential	[15]
*Plasmodium falciparum*	Saturation mutagenesis	Essential	[14]

The genetic models of alternative Sti1/Hop abundance used by Rutledge *et al*. [18] clearly demonstrate the importance of regulated Sti1/Hop levels to maintaining normal cellular function. Reduced levels of Sti1/Hop are detrimental to growth and longevity in yeast, particularly in the context of stress (Fig. 1). This is mirrored by other studies of Hop depletion where cells are still viable but show growth defects, advanced ageing, and reduced stress resilience (Table 1). The suppressed growth with Sti1/Hop overexpression was less expected, particularly in the context of cancer biology, where Hop is often overexpressed, and cells maintain high proliferation rates. This phenotype was mediated by the TPR2B domain, which is the most enigmatic of the Sti1/Hop domains in its binding specificity. Interestingly, this study also confirmed that the TPR2A domain was involved in nuclear localisation as previously shown [19], while the formation of Sti1/Hop‐misfolded protein foci appeared to require the TPR2A and TPR2B domains. Most strikingly, the effects of altered Sti1/Hop abundance were observed even in the absence of Hsp90, confirming a proteostatic function for Sti1/Hop independent of Hsp90 (Fig. 1). This challenges the dogma that suggests Sti1/Hop is simply a scaffold to bring Hsp70 and Hsp90 into proximity and that Sti1/Hop requires a chaperone to interact with client proteins.

**Fig. 1:**
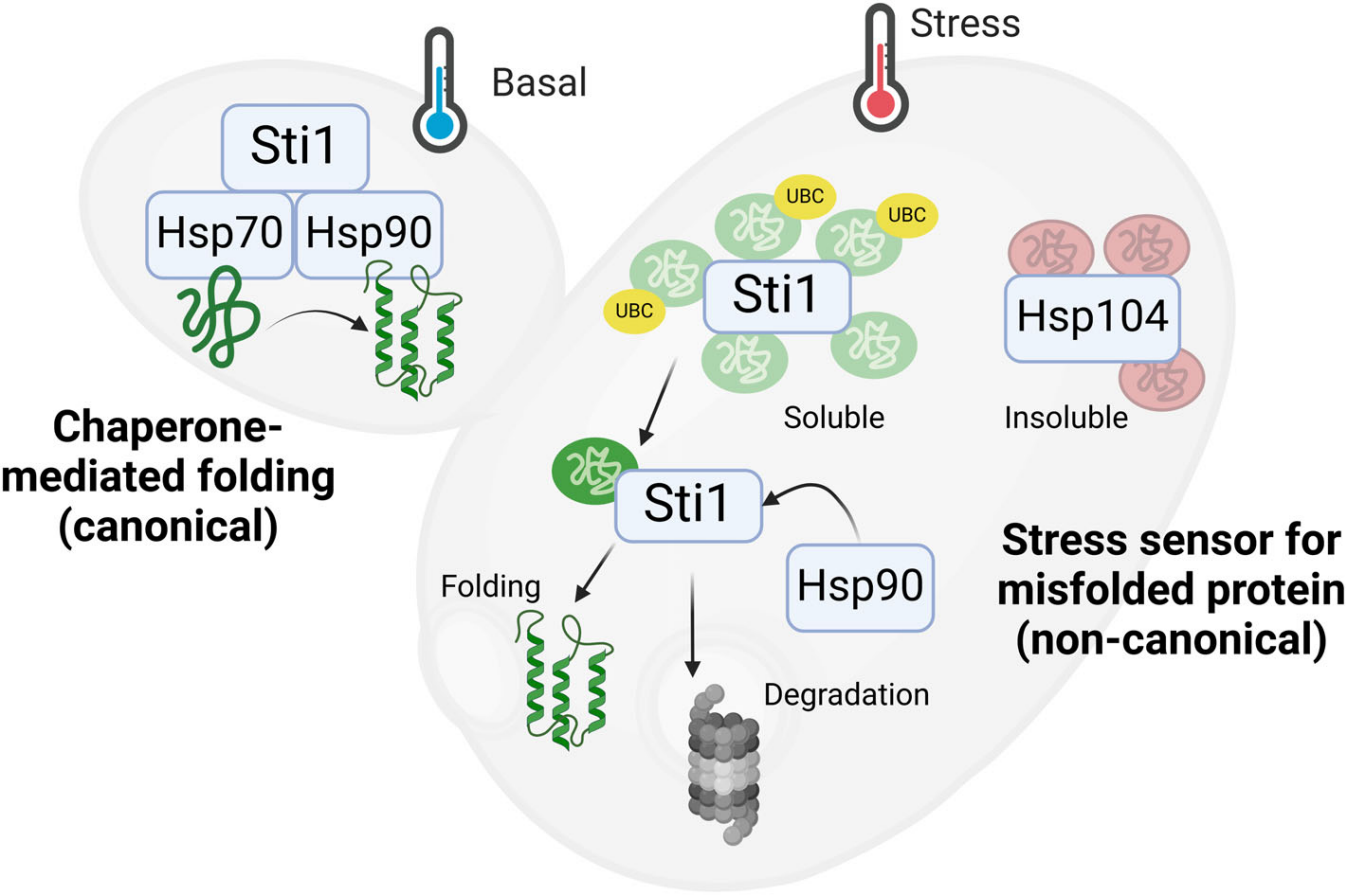
Canonical and non‐canonical roles for Sti1/Hop in proteostasis. Sti1/Hop functions as a cochaperone in the Hsp70‐Hsp90 complex for chaperone‐mediated protein folding under basal conditions. During proteostatic stress, Sti1/Hop sequesters misfolded soluble ubiquitinated proteins in foci independently of Hsp90, although Hsp90 is required for subsequent resolution of foci. This function is independent of the Hsp104 recognition of insoluble protein aggregates.

The thread that emerges from previous reports and is reinforced by the findings of Rutledge *et al*. [18] is the fact that the importance of Sti1/Hop emerges during cell stress (Fig. 1). Sti1/Hop may not always be essential, but all models in which it is depleted show altered stress resilience (Table 1). Perhaps Sti1/Hop is specialised for co‐chaperoning proteostasis during stress? In the study by Rutledge *et al*. [18], either the loss of Sti1/Hop or low overexpression of Sti1/Hop led to activation of the heat shock response (HSR) under basal conditions. In contrast, low overexpression of Sti1/Hop under stress conditions did not induce the HSR. This suggests altered Sti1/Hop levels are sufficient to induce heat shock factor 1 (Hsf1) activation. However, the increased HSR coincided with reduced fitness induced by altered Sti1/Hop levels. Consistent with this, combined overexpression of Sti1/Hop with wild‐type or constitutively active Hsf1 also reduced growth. Therefore, misbalanced Sti1/Hop levels relative to the proteostasis network negatively influence the cellular response to the HSR. Instead of supporting survival, activation of the HSR in an environment in which Sti1/Hop levels are deregulated is deleterious for growth in yeast and is consistent with some mammalian models of Hop depletion.

The implications of the data are that, beyond being a scaffold for Hsp70‐Hsp90 complexes, Sti1/Hop may be a stress sensor that functions to maintain protein solubility and prevent aggregation, much like the holdase function of Hsp70 (Fig. 1). Multiple Hsp70 chaperones may recognise short, high‐frequency hydrophobic sequences in proteins. Binding prevents aggregation, but binding may also stall folding, particularly when Hsp70 levels are high. Therefore, Hsp90 is required to restart folding by promoting the release of misfolded proteins from Hsp70 [8]. The ability of Sti1/Hop to sequester soluble proteins into foci was independent of Hsp90; however, the resolution of these Sti1/Hop‐associated foci after stress required Hsp90, consistent with the model for reactivation of folding by Hsp90 downstream of Hsp70. Sti1/Hop seems to be specialised for soluble aggregates, since the Hsp104 pathway for insoluble proteins is maintained in its absence. Sti1/Hop may therefore function in the immediate stages of stress to segregate soluble misfolded proteins until Hsp90 can be recruited for stabilisation and refolding. This would then suggest a broader role for Sti1/Hop in cells during proteostatic stress, one more analogous with the holdase function of Hsp70 (Fig. 1).

A holdase function for Sti1/Hop needs to be experimentally demonstrated; however, if it were the case, this would be unprecedented for Hsp90 cochaperones. Several JDPs, particularly from the DNAJB class, function as Hsp70 cochaperones in addition to having independent aggregation suppression activity [20]. But this has not been described for any Hsp90 cochaperones, including Sti1/Hop.

## Conclusion

Many questions remain. The identification of the Sti1/Hop soluble, misfolded protein interactome will be interesting. Is Sti1/Hop promiscuous, binding a wide range of misfolded proteins similarly to Hsp70, or is it more selective in its clientele, like Hsp90? Are there domains and associated recognition motifs on Sti1/Hop for proteins other than Hsp70/Hsp90? Does binding occur via a different Sti1/Hop domain (beyond the TPR and DP domains) and is there a motif that is required for Sti1/Hop binding to soluble misfolded proteins? Most importantly, it will be critical to determine if Sti1/Hop possesses independent holdase function and how this is activated during stress. These studies will provide mechanistic insights into the emerging picture of the biological functions of Sti1/Hop, and enhance our broad understanding of the regulation of cellular proteostasis.

## Conflict of interest

The authors declare no conflict of interest.

## Author contributions

GLB and ALE wrote the manuscript.
